# Inference of Large-scale Time-delayed Gene Regulatory Network with Parallel MapReduce Cloud Platform

**DOI:** 10.1038/s41598-018-36180-y

**Published:** 2018-12-12

**Authors:** Bin Yang, Wenzheng Bao, De-Shuang Huang, Yuehui Chen

**Affiliations:** 1 School of Information Science and Engineering, Zaozhuang University, Zaozhuang, China; 2 School of Computer Science and Technology, China University of Mining and Technology, Xuzhou, China; 3 Institute of Machine Learning and Systems Biology, Tongji University, Shanghai, China; 4 School of Information Science and Engineering, University of Jinan, Jinan, China

## Abstract

Inference of gene regulatory network (GRN) is crucial to understand intracellular physiological activity and function of biology. The identification of large-scale GRN has been a difficult and hot topic of system biology in recent years. In order to reduce the computation load for large-scale GRN identification, a parallel algorithm based on restricted gene expression programming (RGEP), namely MPRGEP, is proposed to infer instantaneous and time-delayed regulatory relationships between transcription factors and target genes. In MPRGEP, the structure and parameters of time-delayed S-system (TDSS) model are encoded into one chromosome. An original hybrid optimization approach based on genetic algorithm (GA) and gene expression programming (GEP) is proposed to optimize TDSS model with MapReduce framework. Time-delayed GRNs (TDGRN) with hundreds of genes are utilized to test the performance of MPRGEP. The experiment results reveal that MPRGEP could infer more accurately gene regulatory network than other state-of-art methods, and obtain the convincing speedup.

## Introduction

Inferring gene regulatory network (GRN) is the primary and important biochemical network, which contains the regulatory relationships among genes, proteins and small molecules^1,2^. To infer and analyze gene regulatory network could understand the intracellular physiological activity and function of biology, interaction in the pathway and how to make the organism change^3–5^. Time delay is a very important characteristic in biological regulation mechanism, especially for regulation process^6,7^. The proteins translated by transcription factor (TF) regulate the target gene. This regulation process requires a time lag, which involves the regulation of protein translation, folding, nuclear transport, turnover, and the extension of the target mRNA^8,9^. Thus time-delayed factor is critical to gene regulation process. Inferring time-delayed GRN (TDGRN) is one of the major hotspots in system biology^10^.

To design gene regulatory network modeling methods need to consider time-delayed factor. The time-delayed versions of GRN modeling methods have been proposed. Li *et al*. proposed a unified approach based on time-delayed correlation algorithm for design of time-delayed gene expression matrix and inference of TDGRN^11^. Ngom *et al*. proposed a new extending version of Bayesian network, namely Max-Min high-order dynamic Bayesian network, to model the time lags between TFs and target genes^12^. Chueh and Lu presented a new method based on time-delay Boolean networks to infer biological pathways^13^. Kordmahalleh *et al*. proposed a hierarchical recurrent neural network (HRNN) to identify TDGRN with time series gene expression data^14^.

To understand deeply the specific mathematical relationships between TFs and target genes, differential equation model was proposed to infer GRN^15–18^. Some research added time-delayed factor into differential equation model for TDGRN inference. Chowdhury *et al*. presented time-delayed S-System (TDSS) model to identify simultaneously both instantaneous and time-delayed interactions of TDGRN^19^. But in Chowdhury’s method, differential evolution (DE) algorithm was utilized to optimize all parameters in a TDSS model, and the computing load is very large for the large-scale GRN. In order to reduce computing load, we proposed restricted gene expression programming (RGEP) and particle swarm optimization (PSO) to evolve the TDSS model^20^. This method could select TFs automatically and the number of optimized parameters is reduced greatly. However the execution time is still unacceptable for GRN inference with hundreds of genes^21–23^. Parallel technology is urgently needed to decrease the computing cost of the algorithm.

MapReduce framework as a parallel programming model is utilized for parallel computation over the past few years^24–26^. Recently many methods based on the MapReduce model have been widely applied in various fields, especially in bioinformatics^27–32^. Hu *et al*. presented a modified variable-length associative sequential pattern discovery (VLASPD) method based on MapReduce model for large-scale protein-protein interactions (PPI) forecasting^33^. Abduallah *et al*. proposed a new MapReduce algorithm based on information-theoretic approach to infer GRN in a cloud environment^34^. You *et al*. e presented a parallel support vector machine (SVM) model based on MapReduce framework to predict the large-scale PPI with the information of protein sequences^35^.

In order to decrease the computing cost of large-scale TDGRN identification, this paper proposes a novel MapReduce-based parallel restricted gene expression programming (MPRGEP) algorithm for TDSS model identification. In order to evolve the structure and parameters of TDSS model simultaneously, the structure and parameters are encoded as a chromosome in MPRGEP algorithm. According to partition number, split chromosome population over a cloud computing system’s nodes. At each cloud computing node, sub population is optimized iteratively by a novel hybrid evolutionary method based on gene expression programming and genetic algorithm. Then merge them to save as offsprings.

## Method

### Mapreduce overview

**Figure 1:**
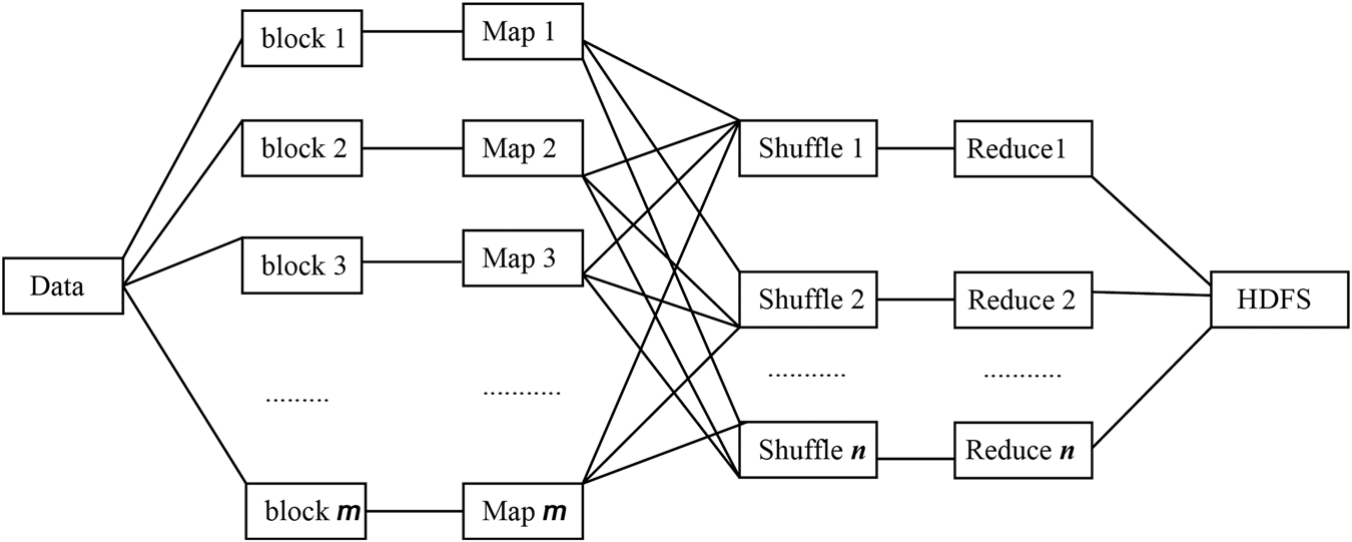
Flowchart of MapReduce framework.

Storage, pretreatment and analysis of biological high-throughput sequencing data have gradually become the main bottleneck of system biology research^36–38^. Hadoop has provided a new solution for big data processing. Hadoop is open-source distributed computing system based on Hadoop Distributed File System (HDFS) and MapReduce framework, and applied to the storage, management and analysis of massive data^39–41^. HDFS is distributed file system, which is utilized to store massive data. MapReduce model is a software framework for big data processing in parallel. MapReduce framework is completed by Map and Reduce operation units, which is described in Fig. 1. In Map phase, input data could be divided into *m* data blocks. Computing nodes calculate Map function in parallel. The pair output $<$key, value> of Map function is stored in each computing node. In Reduce phase, all the intermediate results are combined according to key values and generate the final output, which are stored in HDFS.

### MapReduce-based restricted gene expression programming algorithm

#### Time-Delayed S-system

Due that time-delayed S-system has high accuracy and flexibility, and contains time-delayed factors, which is suitable for modeling time-delayed systems. *t-th* nonlinear time-delayed differential equation in TDSS model is described as followed^42^.

1 $$\frac{d{X}_{i}}{dt}={\alpha }_{i}\prod _{j=1}^{N}{X}_{j,t-{\tau }_{{g}_{ij}}}^{{g}_{ij}}-{\beta }_{i}\prod _{j=1}^{N}{X}_{j,t-{\tau }_{{h}_{ij}}}^{{h}_{ij}},\,i=1,2,\ldots N.$$

Where ${X}_{j,t-{\tau }_{{g}_{ij}}}^{{g}_{ij}}$ is the expression level of gene *X*_*j*_ at $t-{\tau }_{{g}_{ij}}$ time point, ${\tau }_{{g}_{ij}}$ and ${\tau }_{{h}_{ij}}$ are the time-delayed factors, *N* is the total number of genes in TDGRN, *α*_*i*_ and *β*_*i*_ are rate constants of production function and consumption function, *g*_*ij*_ and *h*_*ij*_ are kinetic orders.

#### Chromosome of restricted gene expression programming

**Figure 2:**
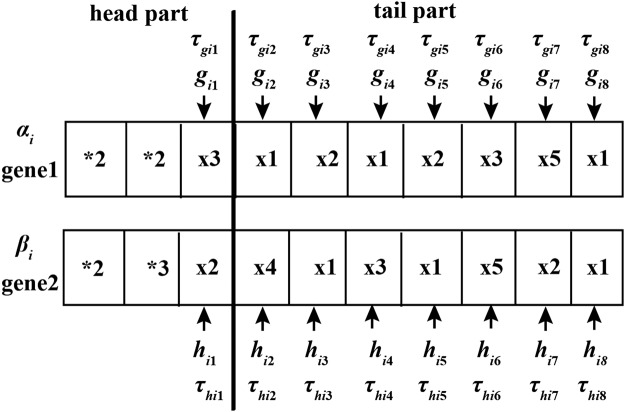
An example of RGEP chromosome with parameters. *I*_1_ is given as $\{{}^{\ast }2,\,{}^{\ast }3\}\cup \{{x}_{1},\,{x}_{2},\,\ldots ,\,{x}_{5}\}.$

In order to better represent and evolve TDSS model, the restricted version of GEP (RGEP) was presented^43^. In RGEP each chromosome of RGEP contains only two genes. An example of RGEP chromosome is described in Fig. 2. The subtraction operator (−) is utilized to connect two genes. Each gene contains head part and tail part, which are created randomly using function set (*F*) and variable set (*T*).

2 $${I}_{1}=F\cup T=\{{}^{\ast }1,\,{}^{\ast }2,\,{}^{\ast }3,\,\ldots ,\,{}^{\ast }n\}\cup \{{x}_{1},\,{x}_{2},\,\ldots ,\,{x}_{m},\,R\}.$$

Where *n represents the multiplication of *n* operands, ${x}_{i}(i=1,\,2,\,\ldots m)$ represents the input variable and *R* denotes constant.

In each gene, the symbols of head part can be selected from set *I*_1_ randomly. The tail part is created randomly with variable set *T* only. In advance, the head length (*h*) is specified for the problems solved. The tail length (*t*) is calculated according to $h$.

3 $$t=(n-1)\times h+1$$

Where *n* represents the largest number of the operands of functions in set *F*.

TDSS model has three kinds of parameters: rate constants (*α*_*i*_ and *β*_*i*_), kinetic orders (*g*_*ij*_ and *h*_*ij*_) and time-delayed factor (${\tau }_{{g}_{ij}}$ and ${\tau }_{{h}_{ij}}$), so we add these parameters into the chromosome in RGEP. As shown in Fig. 2, gene1 and gene2 are given *α*_*i*_ and *β*_*i*_, respectively. In each gene, kinetic order (*g*_*ij*_ or *h*_*ij*_) and time-delayed factor (${\tau }_{{g}_{ij}}$ or ${\tau }_{{h}_{ij}}$) need to be allocated to each terminal node.

**Figure 3:**
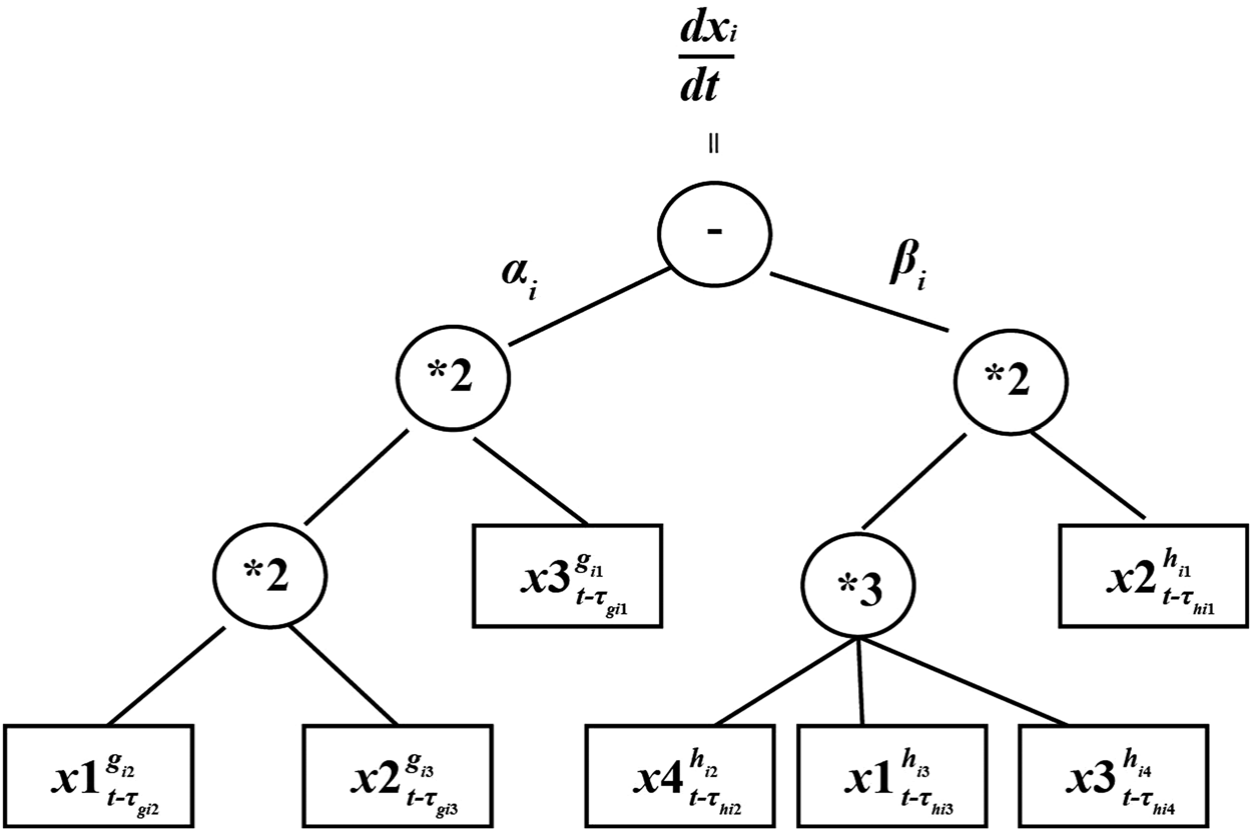
The expression tree of a RGEP chromosome with the parameters.

Figure 3 describes the arithmetic expression trees (ETs) of Fig. 2. Its decoding differential equation expression is shown as follows.

4 $$\frac{d{x}_{i}}{dt}={\alpha }_{i}{x}_{3,t-{\tau }_{{g}_{i1}}}^{{g}_{i1}}{x}_{1,t-{\tau }_{{g}_{i2}}}^{{g}_{i2}}{x}_{2,t-{\tau }_{{g}_{i3}}}^{{g}_{i3}}-{\beta }_{i}{x}_{2,t-{\tau }_{{h}_{i1}}}^{{h}_{i1}}{x}_{4,t-{\tau }_{{h}_{i2}}}^{{h}_{i2}}{x}_{1,t-{\tau }_{{h}_{i3}}}^{{h}_{i3}}{x}_{3,t-{\tau }_{{h}_{i4}}}^{{h}_{i4}}.$$

#### Hybrid evolutionary method

**Figure 4:**
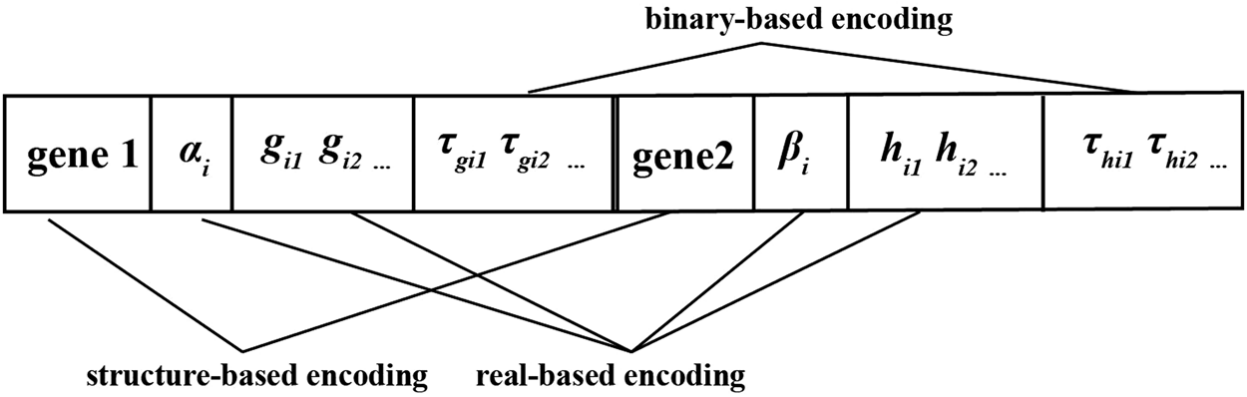
Encoding form of chromosome i in the hybrid evolutionary method.

In order to search the optimal TDSS model, an original hybrid optimization approach based genetic algorithm^44–46^ and gene expression programming^47–49^ is proposed in REGP. The structure and parameters in a TDSS model need to be optimized, which are shown in Fig. 3. In our hybrid evolutionary method, two genes of RGEP and parameters are encoded into one chromosome, which is depicted in Fig. 4.

One chromosome contains three kinds of encoding forms. In Fig. 4, gene1 and gene2 are structure-based encoding, rate constants (*α*_*i*_ and *β*_*i*_) and kinetic orders (*g*_*ij*_ and *h*_*ij*_) are real-based encoding, and time-delayed factors (${\tau }_{{g}_{ij}}$ and ${\tau }_{{h}_{ij}}$) are binary-based encoding. Single evolution strategy could not reach the optimization purpose, so a hybrid evolutionary method is utilized to reproduce the chromosomes.

(1) Mutation. Mutation probability *p*_*m*_ is defined in advance. According to the encoding case, three mutation strategies are utilized, which are introduced as followed.

1. Structure-based mutation

- Single-point mutation. The symbols in the head part could be changed to any symbol, which is selected from set *I*_1_ randomly. The symbols in the tail part can only be changed into a symbol from variable set *T*. Therefore, single-based mutation could create the legal offspring.
- Single-gene mutation. One gene in a chromosome is selected by random, which is replaced by the new gene.
- Change all the variables. All terminal symbols in the structure-coding region are replaced with another terminal symbols.

(2) Real-based mutation
  For each real value $X$ in the real-coding region, create a real value *r* in the interval [0, 1] randomly. If *r* < *p*_*m*_, real value *X* could be mutated with the following Equation.
  5 $$X^{\prime} =X+\delta .$$
  Where *δ* is Gaussian random value.
(3) Binary-based mutation

For each binary value in the binary-coding region, generate a real value *r* in the interval [0, 1] randomly. If *r* < *p*_*m*_, the corresponding binary value is inverted.

(2) Crossover. According to the encoding case, three crossover strategies are utilized. First two parents (*X* and *Y*) are chosen with the crossover probability *p*_*c*_, which is defined in advance.

1. Structure-based crossover

- Single-point recombination. A random point is selected from the structure coding region. Exchange the symbol operators of two parents, which are after this point.
- Single-gene recombination. Two random genes chosen from two parents are swapped.

(2) Real-based crossover
  Two parents (*X* and *Y*) implement crossover operator with following Equation.
  6 $$X^{\prime} =X+\gamma (X-Y).$$
  7 $$Y^{\prime} =Y-\gamma (X-Y).$$
  Where $\gamma =0.99\,{\gamma }^{t}.$ *γ* is a variable related to iteration number *t*. This strategy can change the individuals with a wide range in the early stage of optimization, and protect the better individuals in the later stage.
(3) Binary-based crossover

- Single-point crossover
  A binary point in the binary-coding region is selected randomly. The binary symbols before the point selected are exchanged in order to create the new offsprings.
- Two-point crossover

Select two points in the binary-coding region randomly. The binary string between two points is exchanged between parents.

(3) Selection method. Roulette sampling algorithm is proposed to select the chromosomes to be copied into the next generation according to the fitness values.

#### Flowchart of time-delayed gene regulatory network inference

**Figure 5:**
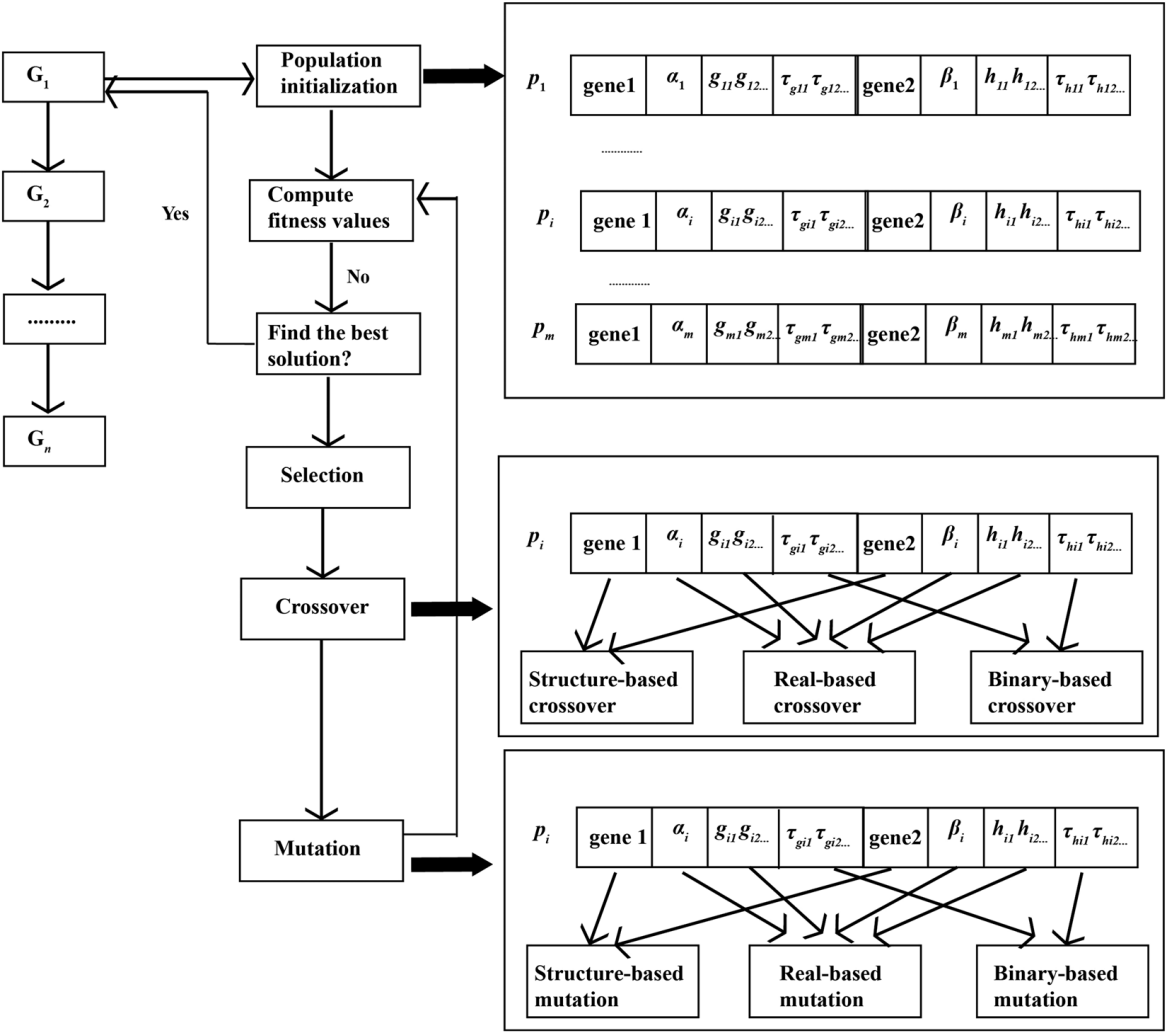
The main flowchart of TDGRN inference.

Inference flowchart of TDGRN with $n$ genes $({G}_{1},\,{G}_{2},\,\ldots ,\,{G}_{n})$ is depicted in Fig. 5. Decomposition strategy is utilized. From *G*_1_ to *G*_n_, regulatory relationships of each gene are identified by optimizing the TDSS models.

1. Initialize population $({p}_{1},\,{p}_{2},\,\ldots ,\,{p}_{m})$ containing structure and parameters. The chromosome structure is described in Fig. 5.
2. The fitness values of all the chromosomes are calculated. If the optimal model is found, stop; otherwise go to (3).
3. The hybrid evolutionary method is utilized to create the offsprings, which is introduced in Section 2.2.3. According to encoding type, select different crossover and mutation strategies. Go to (2).

Through the optimized TDSS model, gain the regulatory relationships of each gene. Finally the regulatory relationships of all genes constitute gene regulatory network.

#### MapReduce-based hybrid evolutionary method

**Figure 6:**
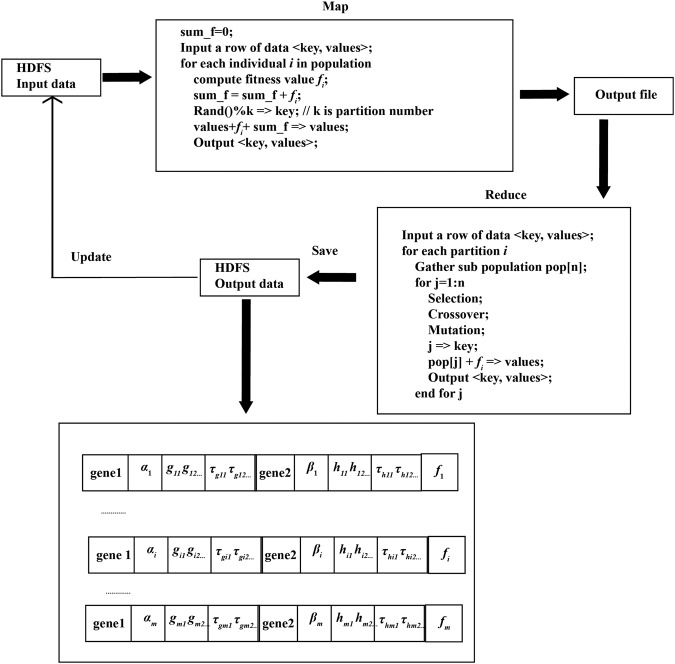
The proposed hybrid evolutionary framework with the Hadoop MapReduce model.

To infer large-scale gene regulatory network and reduce high computation load, our hybrid evolutionary method based on Hadoop MapReduce framework is proposed. This framework distributes evolutionary tasks to Map and Reduce modules. Figure 6 shows the hybrid evolutionary framework with the Hadoop MapReduce model.

1. Input data. The input data are stored on the HDFS, which contain two types of data. The first type of data is chromosome information including the structure and parameters. The second type of data is the fitness value of the corresponding chromosome.
2. Map phase. Each computation node can operate in Map phase independently, without waiting for other nodes. The task of computing node is to calculate the fitness value *f*_*i*_ of the *i-th* chromosome. The fitness values of all chromosomes are accumulated to obtain *sum*_*f* for selection operation. According to the input file, the framework divides the chromosome population into computation nodes (Mappers) in order to achieve parallel computing. In order to realize the crossover operation between chromosomes, we randomly divide the population into different partitions. The chromosomes with the same partition id could implement crossover operator. The number of partitions *k* is defined in advance. The partition id of chromosome $partition\_id$ is generated randomly, which is set as the key output of Map phase. The chromosome, fitness *f*_*i*_ and total fitness value *sum*_*f* are set as the value output of Map stage.
3. Reduce phase. The input data in Reduce phase are from Map phase. After the complete execution of the corresponding Map nodes, the Reduce phase can be executed. In the Reduce phase, the chromosomes with the same $partition\_id$ are collected into a group, obtaining a sub population. The optimization tasks of sub population are distributed to the same computational node (Reducer). With *f*_*i*_ and *sum*_*f*, roulette sampling algorithm is utilized to create the offsprings. The individuals in sub population could implement crossover and mutation operator. The gained sub offsprings and fitness values are written to output file of the Reduce phase in order to update the input data on the HDFS. If the number of iterations reaches the termination condition, the algorithm is terminated; otherwise, go to the Map phase.

## Experiments

Our proposed parallel algorithm MPRGEP is implemented on MapReduce framework. The hadoop version is 2.6.2 and hadoop cluster consists of one master and 30 slaves. The infrastructure hardware of all nodes is comprised of 3.5 GHz Intel Xeon E5–1620 CPU, 4GB DDR2, and Linux CentOS 6.4 (64-bits). The nodes are connected by local area network with transmission speed of 1,000 Mbps. Three criterions are utilized to evaluate the performance of MPRGEP.

8 $${S}_{n}=\frac{TP}{TP+FN}.$$

9 $${S}_{p}=\frac{TN}{FP+TN}.$$

10 $$Speedup=\frac{runtime(Single\,node)}{runtime(cluster)}.$$

**Figure 7:**
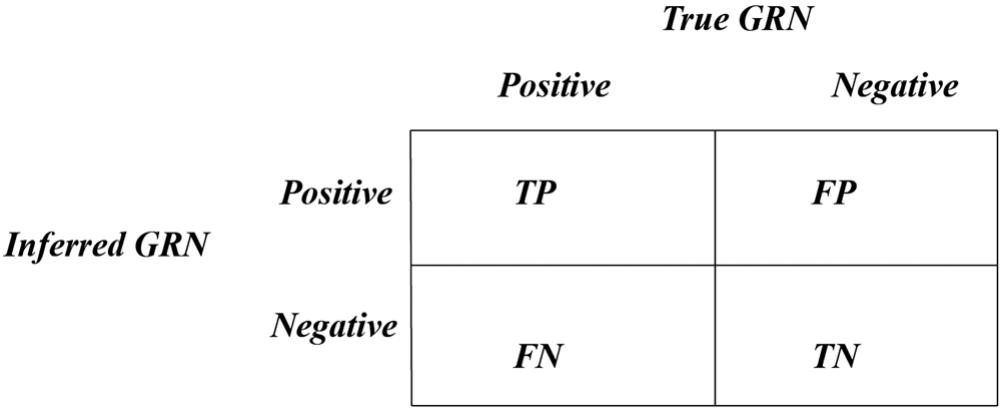
Description of *TP*, *FN*, *FP* and *TN*.

Where *TP*, *FN*, *FP* and *TN* are presented in Fig. 7.

### Artificial dataset

**Figure 8:**
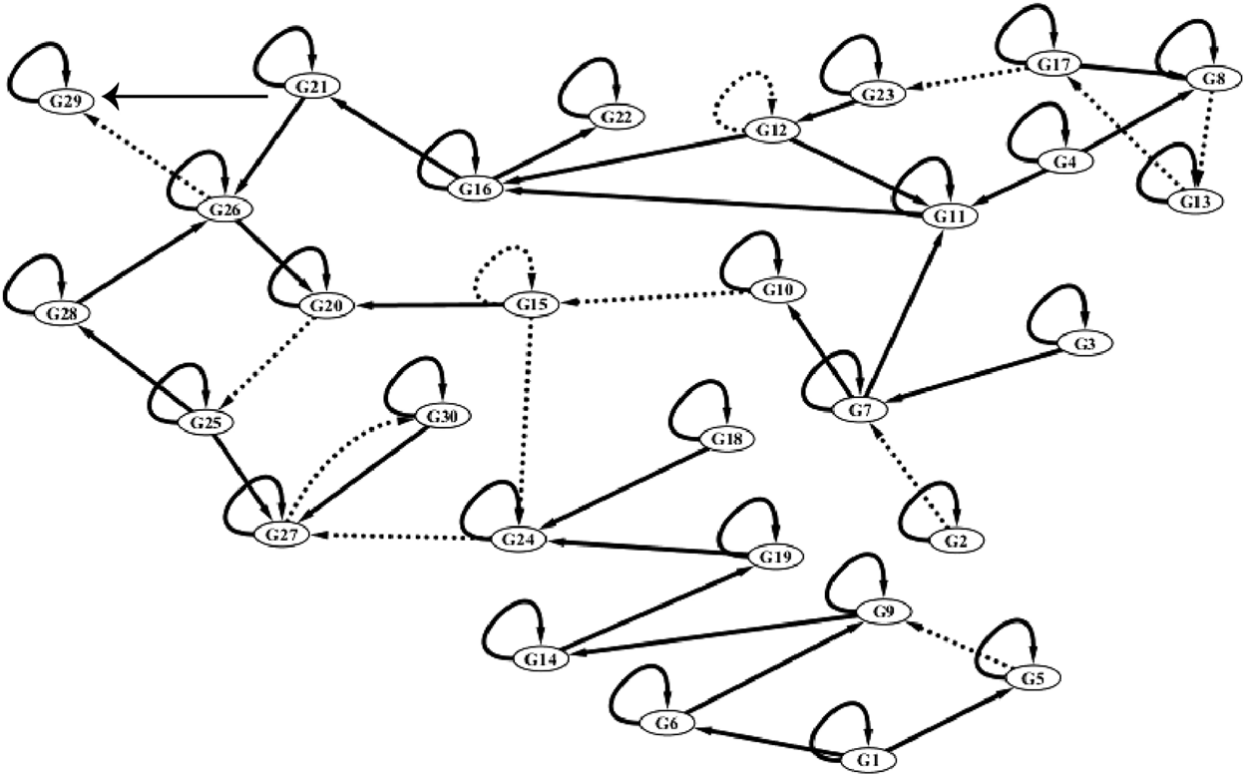
The reconstructed GRN with 30 genes. Solid lines denote the instantaneous regulatory relationships, while dashed lines denote the time-delayed regulatory relationships.

In this part, the parameters of experiments are shown in Table 1, which are selected empirically. The used function set is {*2, *3, *4, *5}. The first artificial dataset is from a 30-gene time-delayed GRN, which is shown in Fig. 8 ^19,20^. Kimura’s method (S-system model based on decomposition strategy and a cooperative coevolutionary algorithm)^21^, DBN (dynamic Bayesian network learned by the likelihood maximization)^22^ and TDSS (time-delayed S-system model based on PSO)^23^ are also applied for 30-gene artificial TDGRN identification. The averaged performance results of four inferred algorithms are represented in Table 2. From Table 2, it could be seen that MPRGEP has a higher sensitivity (*S*_*n*_) than other three methods, which reveals that our method can infer more true-positive regulatory relationship. MPRGEP could identify less false-positive regulatory relationships.

**Table 1:** Parameters in this experiment.

Parameters	Values
Population size	2000
Maximum iteration number	200
Crossover probability *p*_*c*_	0.7
Mutation probability *p*_*m*_	0.3
Rate constants (*α*_*i*_ and *β*_*i*_) interval	[0, 3]
Kinetic orders (*g*_*ij*_ and *h*_*ij*_) interval	[0, 1]
Time-delayed factor (${\tau }_{{g}_{ij}}$ and ${\tau }_{{h}_{ij}}$) interval	[0, 3]
Partition number *k*	200

**Table 2:** Experiment results for 30-gene artificial TDGRN.

	Kimura’s method^21^	DBN^22^	TDSS^23^	MPRGEP
*S*_*n*_	0.8588	0.5662	0.9485	0.971
*S*_*p*_	0.7096	0.9280	0.9832	0.9832

The open-source software GeneNetWeaver 3.1 is utilized to generate three yeast S.cerevisiae sub gene regulatory networks with 50 genes, 100 genes and 150 genes, respectively. Time-delayed regulatory relationships are created randomly and time-delayed values are selected from [0, 3]. Three time-delayed gene regulatory networks are described in Table 3. Initial conditions are randomly generated. For each network, 10 time-series datasets are generated and each dataset contains 21 time points from 0 to 20.

**Table 3:** Description of three time-delayed gene regulatory networks.

Number of genes	Number of regulatory relationships	Number of time-delayed regulatory relationships
50	128	10
100	212	16
150	345	25

Our method is executed in the single machine and computing clusters with 20 computing nodes, respectively. Through several runs, the averaged performances are listed in Table 4. From the inference results, we know that MPRGEP not only can solve large-scale time-delayed gene regulatory network, but also perform well in terms of *S*_*n*_ and *S*_*p*_. Table 4 also reveals that MapReduce framework could reduce running time of GRN inference, which makes it possible to identify large-scale GRN with more genes.

**Table 4:** Performance of three TDGRNs by running MPRGEP.

Method	MPRGEP with single node			MPRGEP with computing cluster		
	*S*_*n*_	*S*_*p*_	Runtime (*m*)	*S*_*n*_	*S*_*p*_	Runtime (*m*)
50 genes	0.5781	0.9483	172.3	0.5859	0.9432	44.5
100 genes	0.533	0.9237	728.7	0.5283	0.9223	115.7
150 genes	0.469	0.905	1192.6	0.4753	0.909	159.0

**Figure 9:**
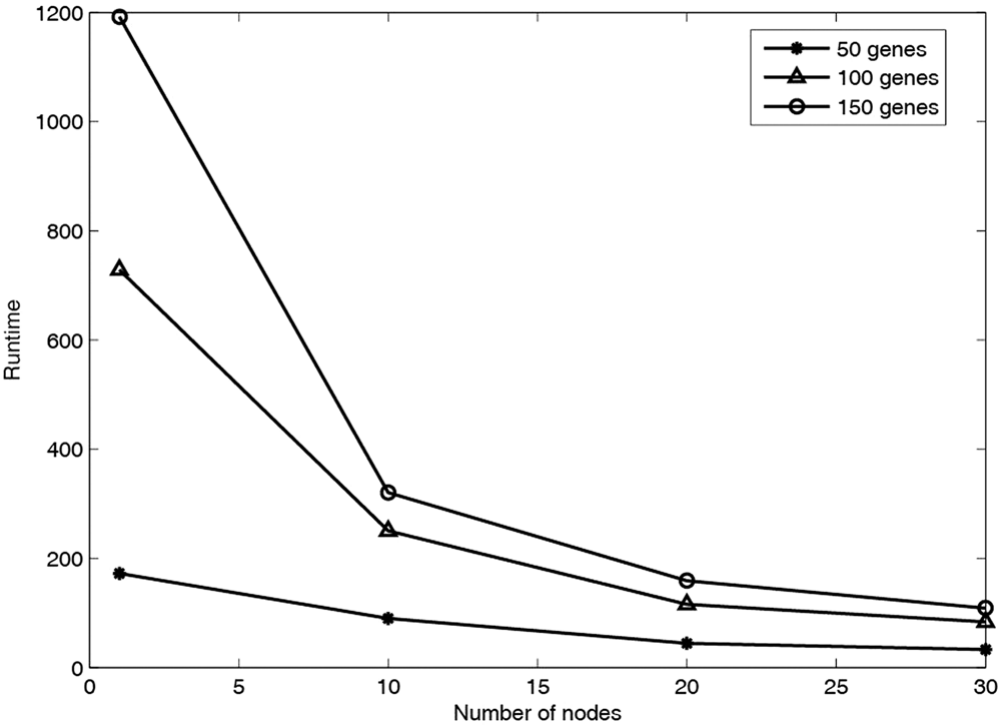
Runtime performance of MPRGEP for three TDGRNs inference.

**Figure 10:**
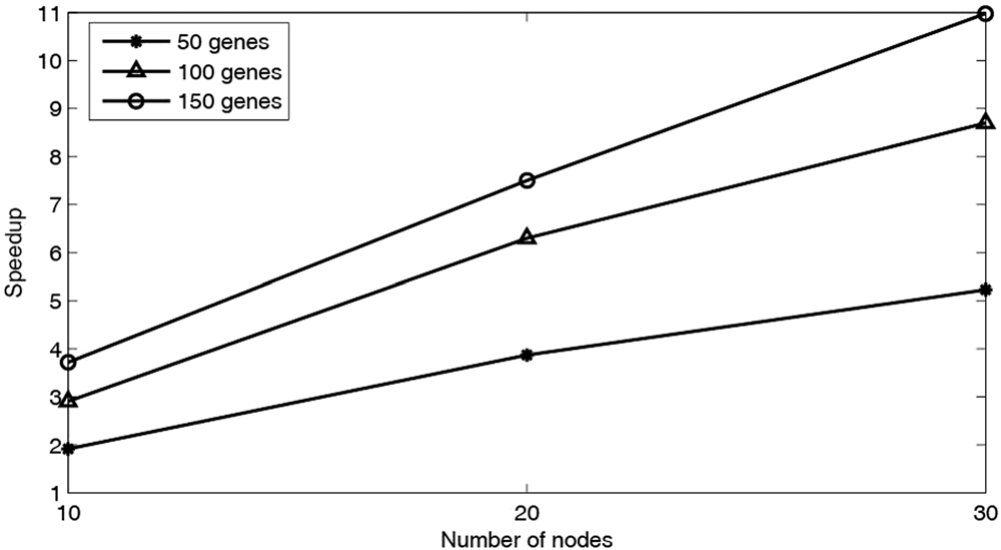
Speedup performance of MPRGEP for three TDGRNs inference.

In order to validate the parallel computing performance, MPRGEP algorithm is utilized to identify three above time-delayed GRNs in three computing clusters with 10, 20 and 30 nodes, respectively. The runtime and speedup performance are depicted in Figs 9 and 10. From Fig. 9, it could be seen clearly that as the number of genes rises, the running time also rises. With the increment of computing nodes, the running time decreases. Figure 10 shows that as the number of computing nodes increases, our proposed parallel algorithm accelerates significantly. The best speedup performance of MPRGEP is the case that MPRGEP is run on 30 computing nodes to infer GRN with 150 genes. The speedup curve is not linear because of serial bottlenecks and infrastructure barriers in MapReduce framework.

**Figure 11:**
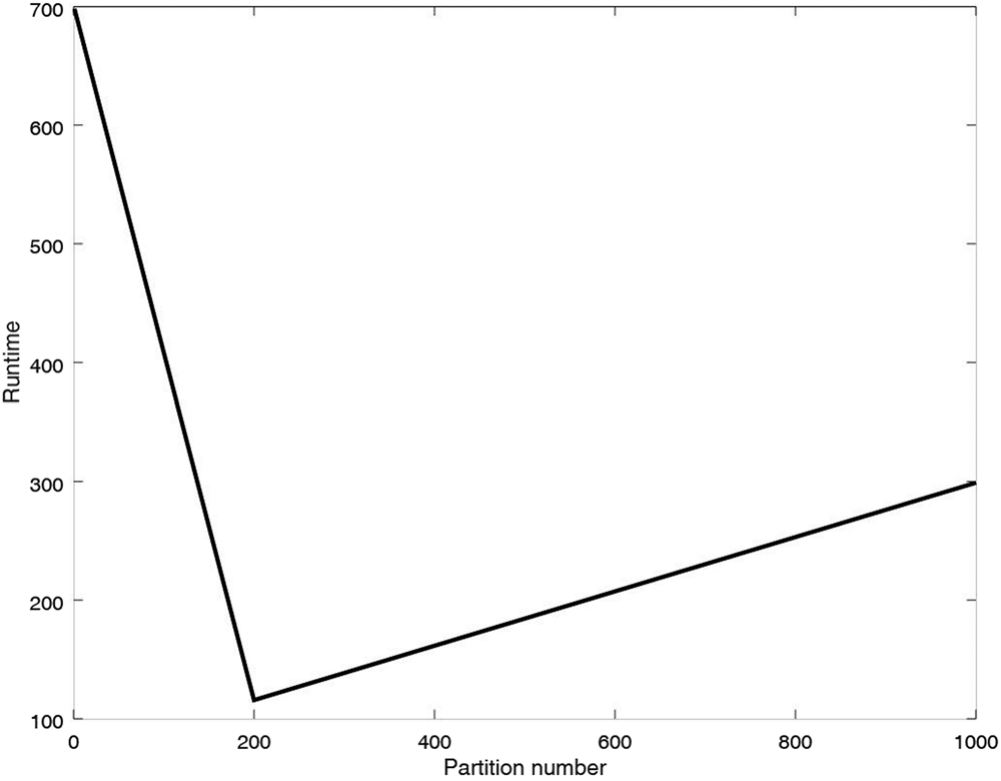
Performance of MPRGEP with different partition numbers.

In MPRGEP, the computational tasks of hybrid evolutionary algorithm are mainly concentrated in the Reduce phase. The sub population with the same partition id will be assigned to the same Reduce for optimization. If the number of Reducers is fixed in advance, the number of partitions can affect the speed of parallel computation. We make the experiments with three partition numbers, 1, 200 and 1000. Node number in the computing cluster is set as 20. The running time is depicted in Fig. 11. From the result, we can see that the hybrid evolutionary algorithm performs best when partition number is set as 200. When the partition number is 1, the sub population contains all the population and is optimized in one Reducer. Parallel strategy doesn’t work. When the number of partition number is given to 1000, the number of sub populations is too large. In this case, more Reducers are needed. The allocation and merging of resources could waste more time.

### Real biology dataset

**Figure 12:**
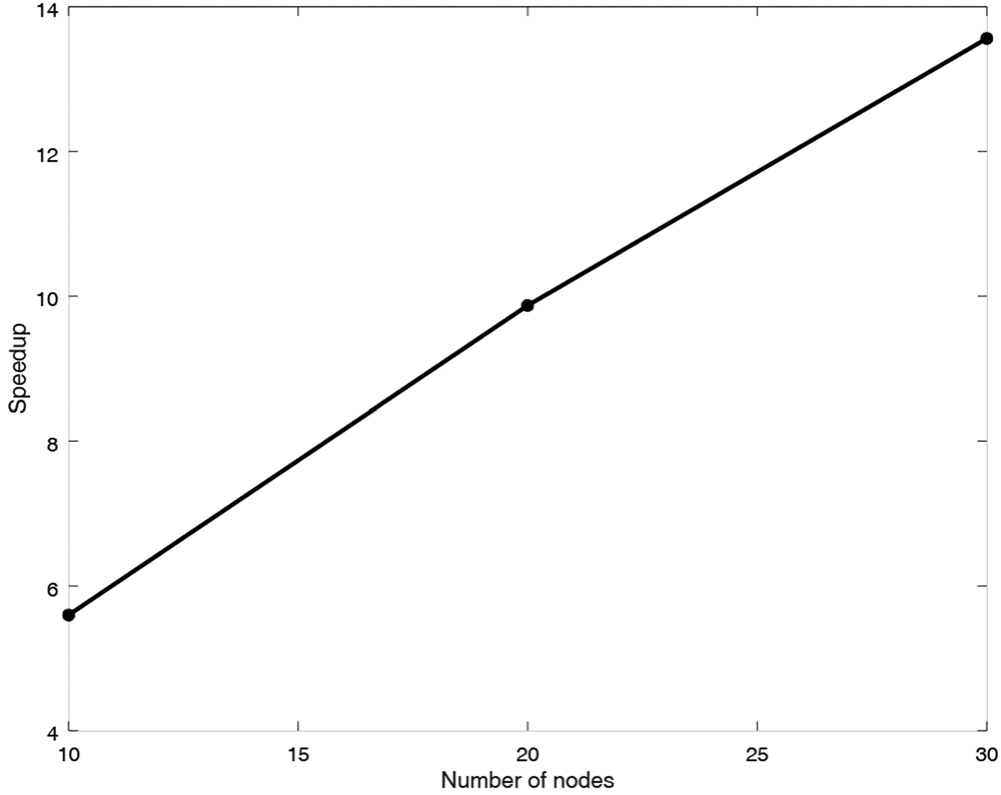
Speedup performance of MPRGEP for GRN inference with 500 genes.

In this part, the dataset is from the Gene Expression Omnibus (GEO) at http://www.ncbi.nlm.nih.gov/geo/ (GEO accession: GSE30052)^34,50^. This dataset contains 5,744 probe sets, 10,928 genes and 49 time points. In order to validate the parallel performance of MPRGEP, one subset from this dataset is extracted, containing 500 genes. The experiment is executed in the computing clusters with 20 nodes. The parameters are also from Table 1. The running results are described in Fig. 12. From Fig. 12, it can be seen that our method could be accelerated evidently.

## Discussion and Conclusion

With the rapid development of biotechnology, gene regulatory networks inferred contain more genes, so there is necessity for developing advanced computational algorithm to infer gene regulatory network with gene expression data. This paper proposes time-delayed S-system model to model instantaneous and time-delayed regulation interactions in time-delayed gene regulatory network. A novel MapReduce-based parallel restricted gene expression programming (MPRGEP) algorithm is utilized for TDSS model identification. The experiment results reveal that our parallel algorithm is promising in terms of accuracy and speedup when used to infer large-scale TDGRN.
